# Practical Departments

**Published:** 1905-09-09

**Authors:** 


### PRACTICAL DEPARTMENTS.

### THE AMERICAN RADIATOR COMPANY'S HEATING APPARATUS.

The American Radiator Company, whose headquarters on this side are at 89 and 90 Shoe Lane, London, E.C., make a very varied assortment of apparatus for the heating of buildings, by either hot water or steam. Their speciality, as their name implies, is the radiator, of which they make a great many types—with a single row of vertical columns for the hot water or the steam, with two rows, three, or four rows. They make also types known as the Verona, the Tuscan Flue, the Rococo, and the Italian ornamental flue, all in differently ornamental styles; the Italian plain flue, wall radiators, hospital pattern radiators, hinged radiators, curved radiators, etc. The different forms of radiators are intended for different situations and different conditions. The two-column apparatus, for instance, gives not quite twice the heating surface of the single-column, and the four-column, again, not quite twice that of the two-column. The apparatus with Italian names have also a larger heating surface than those with English names. By heating surface is meant the extent of surface of the radiator, measured in square feet, that is radiating heat, sending out heat waves into the surrounding space. As with other firms the company arrange to combine heating with ventilation, and have therefore worked out different forms of louvres, inlet valves, and other apparatus to go with the radiators, and to be fixed in the floor, in the wall, the ceiling, or wherever may be convenient. In connection with the ventilation, they arrange adjustable plates to be fixed on one side of the radiator, to direct the cold entering air up through the radiator, and so to pass over a larger portion of the heated surface than it would do if it passed straight through between the columns.

Two Special Forms of Radiator.

The great point sought by the designer of apparatus intended either to receive heat, or to distribute it, is large surface, in as small a space as possible, and to accomplish this the American Radiator Company have designed two forms of radiators, both to be fixed in a horizontal position, the columns lying down instead of standing up. In one form—"The Excelsior"—the surfaces of the pipes of which the apparatus is formed are corrugated, like the galvanised iron plates that are so much used for temporary buildings. In the other—"The Sanitary Pin"—apparatus, each section is in the form of two flat plates with a space between them—a flat box, in fact—both top and bottom plates having a number of pins each projecting 1 inch from the metal plate of which it forms a part, and with which it is cast. All the radiators are arranged to be put together in sections, each section exposing a certain surface, so that any required surface can always be arranged. The company make "Dining Room" radiators, having hot-closets forming part of the apparatus, that should be very useful in many situations besides dining-rooms. They also make boilers for heating purposes on the sectional plan, both for heating water and for generating steam; as well as storage tanks for hot water, arranged for delivering steam directly to the water, or for heating the water by passing steam through pipes carried in the tank, and again for connecting directly to a hot-water boiler. The company make and keep stocks of every kind of valve and connecting piece and their accessories. They make a pressure-reducing valve that should be useful where live steam is employed for heating water, back-pressure valves, and a special form of nozzle for regulating the flow between the main and branch pipes, and, in fact, every necessary adjunct for heating supply. Where there is a fittingshop at the hospital, the pipe machines they supply for cutting and threading pipes should be of service. It is often a great advantage to be able to execute repairs quickly on the premises.

### THE STREBEL BOILER FOR HEATING WATER.

Hot water is required in large quantities for general use in every hospital, and it is also used very much for heating purposes in connection with hot-water radiators. There are two methods by which the water used for either purpose may be heated—viz., by the direct action of the heat generated by the combustion of coal, in a boiler, arranged only to heat the water to a certain temperature and not to generate steam; and by utilising the latent heat of steam generated in a steam boiler, in the apparatus known as a calorifier. The Strebel boiler is designed for the former purpose. It is of the type known as sectional; that is to say, the boiler is built up of a number of sections, each of which is a small boiler in itself, arranged to be fitted together in any required number, according to the heating surface required and the quantity of water to be heated.

The fire-box or furnace is placed near the bottom of the space inside the annulus, forming the water space, the fuel resting on hollow fire-bars, through which the water that is being heated also circulates. The fire-bars themselves add to the surface through which heat passes directly to the water. Keeping the bars cool in this way also allows powerful fuels, such as anthracite coal, to be used, and it adds to the life of the bars themselves.

The boiler is constructed to burn anthracite coal and coke, but it may also be used with wood, peat, etc., bearing in mind that the calorific value of these fuels is lower than that of coal. A modification of the boiler is also made for steam generation, a steam drum being fixed above the boiler proper. Messrs. R. O. Meyer and Company, Norfolk House, Norfolk Street, W.C., are the agents in this country.

